# When disasters strike the emergency department: a case series and narrative review

**DOI:** 10.1186/s12245-021-00372-7

**Published:** 2021-09-09

**Authors:** Dennis G. Barten, Vincent W. Klokman, Sigrid Cleef, Nathalie A. L. R. Peters, Edward C. T. H. Tan, Arjen Boin

**Affiliations:** 1 Department of Emergency Medicine, VieCuri Medical Center, P.O. Box 1926, 5900 BX Venlo, The Netherlands; 2 Department of Emergency Medicine, Laurentius Hospital, Roermond, The Netherlands; 3 Department of Emergency Medicine, Radboud University Medical Center, Nijmegen, The Netherlands; 4 Department of Trauma Surgery, Radboud University Medical Center, Nijmegen, The Netherlands; 5 Department of Political Science, Leiden University, Leiden, The Netherlands

**Keywords:** Emergency department, Disaster medicine, Emergency preparedness, Internal hospital disasters, Natural disasters, Manmade disasters, Safety, COVID-19, Pandemic, Terrorism, Cyberattack

## Abstract

**Introduction:**

Emergency departments (EDs) are reasonably well prepared for external disasters, such as natural disasters, mass casualty incidents, and terrorist attacks.

However, crises and disasters that emerge and unfold within hospitals appear to be more common than external events. EDs are often affected. Internal hospital crises and disasters (IHCDs) have the potential to endanger patients, staff, and visitors, and to undermine the integrity of the facility as a steward of public health and safety. Furthermore, ED patient safety and logistics may be seriously hampered.

**Methods:**

Case series of 3 disasters within EDs. Narrative overview of the current IHCD-related literature retrieved from searches of PubMed databases, hand searches, and authoritative texts.

**Discussion:**

The causes of IHCDs are multifaceted and an internal disaster is often the result of a cascade of events. They may or may not be associated with a community-wide event. Examples include fires, floods, power outages, structural damage, information and communication technology (ICT) failures, and cyberattacks. EDs are particularly at-risk. While acute-onset disasters have immediate consequences for acute care services, epidemics and pandemics are threats that can have long-term sequelae.

**Conclusions:**

Hospitals and their EDs are at-risk for crises and their potential escalation to hospital disasters. Emerging risks due to climate-related emergencies, infectious disease outbreaks, terrorism, and cyberattacks pose particular threats. If a hospital is not prepared for IHCDs, it undermines the capacity of administration and staff to safeguard the safety of patients. Therefore, hospitals and their EDs must check and where necessary enhance their preparedness for these contingencies.

## Background

Hospitals, and their emergency departments (EDs) in particular, are crucial elements in the disaster response chain anywhere in the world. These facilities are reasonably well prepared for external events, such as natural disasters, mass casualty incidents, and terrorist attacks [1]. However, if a hospital itself is struck by disaster, it faces different yet difficult challenges. Although internal disasters appear to be more common than external events [2, 3], this topic receives limited attention in medical literature.

Internal hospital crisis and disasters (IHCDs) are defined as sudden onset events that severely disrupt the everyday, routine services of a hospital facility. In popular parlance, these are also referred to as “major incidents within hospitals” [3–5]. ICHDs have the potential to endanger patients, staff, and visitors, and to undermine the integrity of the facility as a steward of public health and safety [1, 3]. Examples include power outages, fires, floods, structural damage, information and communication technology (ICT) failures, and cyberattacks [1, 2, 6].

Hospitals are fertile ground for catastrophic events. They are packed with flammable gasses, toxic substances and biological as well as radioactive material. Simultaneously, hospitals shelter a high number of vulnerable and dependent individuals who are also “strangers” to the building and the organization [1, 7]. Furthermore, healthcare technology is rife with software and hardware flaws, leading to vulnerability [6]. It has been estimated that the annual likelihood of an IHCD in US hospitals lies somewhere between 0.33 and 31%. However, reliable data are lacking [3]. In a nationwide study from the Netherlands, 134 IHCDs were identified between 2000 and 2020. In this same period, the number of hospitals decreased from 107 to 83. Involvement of the ED was reported in 82% of cases, and ED closure could often not be avoided [8].

Dependent on the reach of the disaster, ICHDs may affect the ED in three different ways. First, an IHCD can occur in an isolated fashion within the ED [9, 10] or be part of a multi-departmental event. Second, in case of a crisis or disaster in the same institution, the ED may serve as the focus for disaster operations and victim intake. Third, EDs may be faced with community-wide external events, including catastrophic natural or manmade disasters, that affect the services of a hospital [2, 11–14].

Adequate preparation is the best way to provide resilience for EDs to deal with disaster. This entails recognizing and addressing common problem areas as well developing a thorough disaster plan that is practiced [2]. In this manuscript, we report a narrative review on three disasters having occurred within EDs. It may therefore provide a blueprint to achieve better ED preparedness for IHCDs.

## Case series

### ED ceiling collapse

Date: May 18, 2017; 11:50 p.m. Teaching hospital, level 2 trauma center with 569 beds and 24/7 ED. Disaster type: structural collapse.

**Fig. 1:**
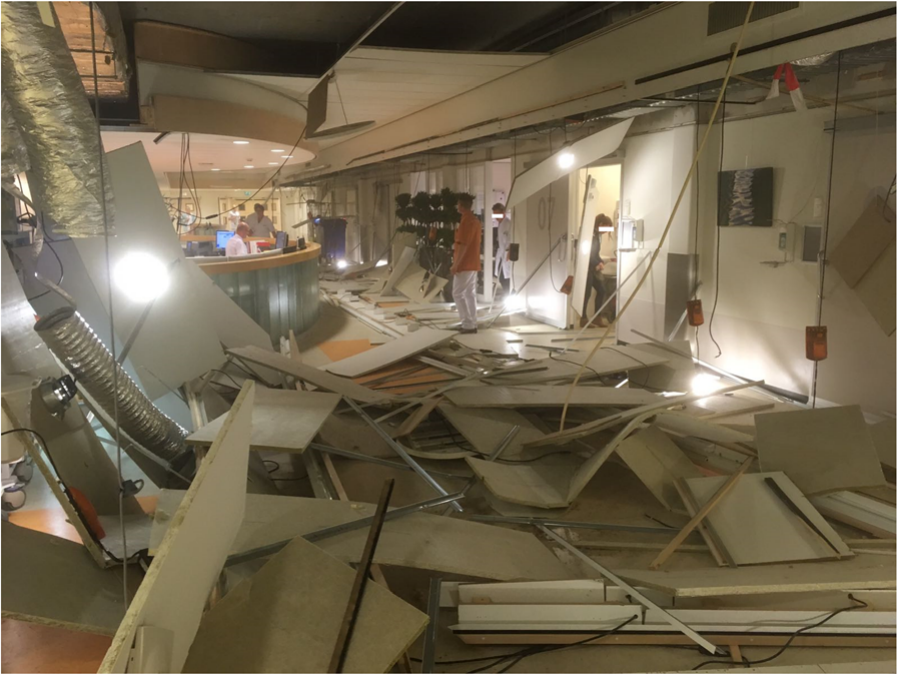
Emergency department ceiling collapse due to construction failure

A construction flaw resulted in the collapse of 75% of the suspended ceiling of the ED of a teaching hospital on a Thursday evening (Fig. 1). No deaths or injuries were reported. The ED was closed immediately and had to be evacuated. This affected two patients and their caregivers; evacuation was completed within 10 min. Ambulances were diverted to surrounding hospitals. In the meantime, the intensive care unit (ICU) was prepared to serve as temporary location for assessment and resuscitation of unstable patients. Simultaneously, the neighboring acute medical unit was transformed into a temporary ED. As a result of this strategy, emergency care could be resumed 8 h after the incident, with the exception of patients in the highest triage category. Another 9 h later, an emergency corridor from the ambulance hallway to the 2 trauma units was completed. From then on, it was possible to treat all patient categories again. It took 6 business days to reconstruct the ED ceiling and to resume emergency care in a normal manner. During this period of crisis, patient safety was maintained and no critical incidents were reported. This event was comprehensively described in a published field report [9].

### Fire in the ED

Date: July 20, 2018; 11 a.m. Community hospital, level 3 trauma center with 290 beds and 24/7 ED. Disaster type: internal fire.

A patient was transferred from the psychiatric ward to the ED on a Saturday morning, following intentional ingestion of toiletry items. At that moment, the psychiatric service judged she was not suicidal. She was placed in one of the patient rooms on the ED, with the door closed, and treatment was started. While left alone in her room for a short while, the patient managed to set fire to the mattress with her clothes also catching fire. Because there was a direct view from the nurses’ station into the patient’s room, the fire was noticed instantaneously and simultaneously with the automatic activation of the fire alarm. The patient was immediately moved out of the room and placed under a shower, followed by appropriate treatment of her severe burn injuries which necessitated transfer to a tertiary burn unit. The immediate closure of the door confined the fire to this one room. Nevertheless, due to the spread of smoke throughout the ED, the department had to be evacuated. Fortunately, this event took place on an exceptionally quiet Saturday morning, when only 3 other patients resided in the ED. Evacuation of these patients was completed within 10 min. Ambulance services and surrounding hospitals were notified about the ED closure.

**Fig. 2:**
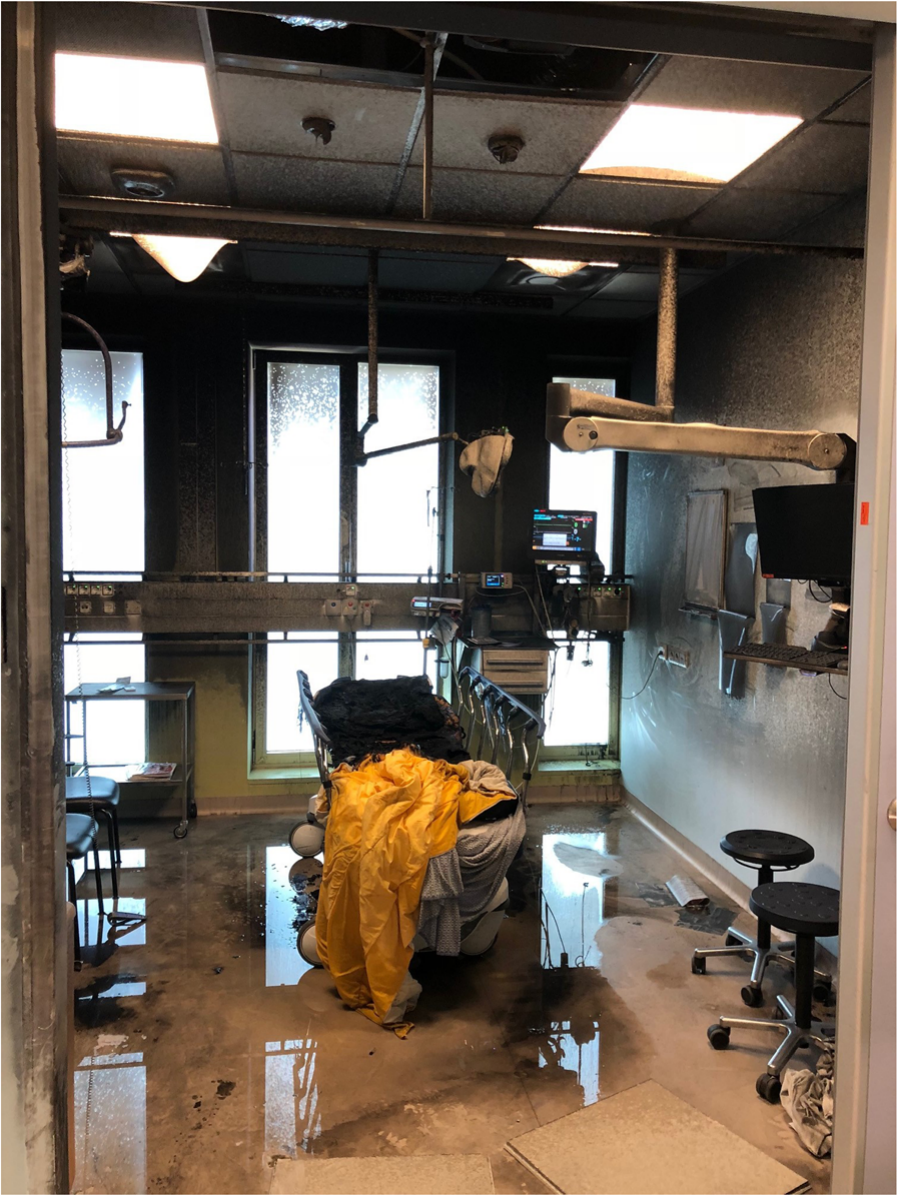
Extensive damage to an emergency department room caused by a fire in the ED

The acute medical unit—situated directly adjacent to the ED—was saved from evacuation due to the compartmentalized design of the building. The operation rooms (ORs) are situated in the floor above the ED; where the smoke alarms went off, necessitating evacuation. At that particular moment, an emergency operation was due to commence any minute. This patient was urgently transferred to another hospital without any further complications. The ED room where the fire had started was completely destroyed (Fig. 2). The rest of the department was seriously affected by the smoke. After the fire brigade declared the ED safe, a specialized cleaning company cleaned the department. It was decided that the acute medical unit should function as alternative ED. Furthermore, the cardiac emergency department and the ICU were prepared to serve as temporary locations for assessment and resuscitation of unstable patients. Three hours after the fire started, delivery of emergency service was started at the alternative locations. At 11 p.m. cleaning of the ED was completed, after which emergency care could be resumed at its primary location. All ED professionals were debriefed by a specially trained professional.

### Computer system failure

Date: January 26, 2018; 10:30 a.m. Academic hospital, level 1 trauma center with 1065 beds and 24/7 ED. Disaster type: Computer system failure.

As a result of a malfunctioning hardware component, a failure occurred in various ICT systems of an academic hospital and level one trauma center on a Friday morning. This included defective network drives, malfunctions in the image processing systems, failure of the closed-circuit television (CCTV; camera system), and loss of wireless internet. The ORs, diagnostic laboratories, and pharmacy departments experienced problems with business continuity. Later, additional digitalized systems were discovered not to work; these included printing services, radiology department image storage, and the internal employee alert systems. Initially, only the pharmacy department was closed. However, due to the possible inability to provide sufficient and safe care to all critical patients, it was decided to have a complete ED patient stop, in order to sustain good and adequate care for all in-hospital patients. A complete stop was announced from 4:30 p.m. to 9:30 p.m. until all ICT systems were up and running again. This was the first ED closure in the history of this hospital.

## Discussion

IHCDs have the potential to occur at any time, at any location, and in any hospital. EDs are at particular risk [8]. The causes are multifaceted and an internal disaster is often the result of a cascade of events [1, 3, 8, 13]. In addition, IHCDs are typically further complicated by failure of communication systems, power outage, water loss, physical damage to the facility, hazardous material exposure, evacuation-related problems, resource allocation difficulties, and problems for additional staff reaching the facility [1, 2]. Although it is impossible to be prepared for each and every crisis, it is imperative to know about the most common types of disaster that may affect the ED. Specific hospital characteristics and its location may determine the prioritization of certain hazards [1].

In the response to disasters, the ED serves multiple roles; from decontamination and triage to stabilization and initial treatment of victims, while continuing to provide routine medical care [2]. These roles are potentially hampered if the department is involved in the disaster itself. There may be physical damage to the ED or to other essential hospital departments, such as the ICU, OR, laboratories, and the radiology department. Triage, treatment, and logistic operations may be further obstructed by power outages and loss of water supply, medical gasses, and ICT systems [1, 13].

### Disaster types

IHCDs can be differentiated in two major groups: internal disasters (the event is confined to the healthcare facility only) and combined internal and external disasters (the healthcare facility is involved in the external event, which may be community-wide). The latter group can be subdivided into natural disasters and manmade disasters [1]. A classification of IHCDs is shown in Table 1. Following external disasters, a significant increase in ED volume of patients should be expected, even if the hospital itself is incapacitated [2, 12]. External disasters may also simultaneously affect multiple hospitals, increasing stress on remaining facilities [13, 15–18].

**Table 1:** Classification of internal hospital crises or disasters

**Internal disasters**	
Internal fire Internal flood Structural damage Power failure Water loss Loss of medical gasses Elevator failure ICT failure Chemical spill Radiation accident Violence Terrorist attack*	
**Combined external and internal disasters**	
**Natural disasters**	
**Geophysical** Earthquake Volcanic eruption Tsunami Landslide **Climate-related** Storm, hurricane, tornado Flood Wildfire Lightning strike **Infectious disease outbreak** Epidemic ~ pandemic	
**Manmade disasters**	
Nuclear and radiation accident Chemical spill Infrastructural disaster Warfare Terrorist attack*	

*Can either be an internal or combined external and internal disaster

### Internal disasters

An internal disaster refers to a sudden-onset event that disrupts the everyday, routine services of the facility, and which is not caused by an external event [1].

#### Internal fires

Although severe fires are comparatively rare, there were 10.662 reported fires in UK hospitals in the decade 1994-2004. The fires caused 4769 patients to be evacuated, and resulted in 651 injuries and 17 fatalities [19]. Most of the hospital fire reports focus on ICUs or ORs, where flammable medical gasses likely play a substantial role and may contribute to explosion hazards [20]. Another fire risk to hospitals may be patients who display risky behavior and/or suffer from suicidal ideations, which was the case in the ED fire described earlier in this manuscript. Fires in hospitals are particularly worrying because smoke can quickly travel within large buildings, aided by air-conditioning and ventilation systems [20].

#### Structural failure

Structural damage of hospitals is mostly caused by external events, such as earthquakes, floods, hurricanes, or catastrophic manmade disasters. However, construction flaws may lead to safety risks as well, as shown by the previously reported ED ceiling collapse [9].

#### Power outage

Power failures are relatively common, although loss of power is only a disaster when auxiliary power sources fail. Hospitals nowadays are highly dependent on electricity, and a power outage typically triggers a cascade of events, including problems with critical care equipment, elevators, temperature regulation, and several ICT systems. Also, communication systems may be switched off [2, 13]. Deaths as a result of hospital power failures have been reported [2]. Therefore, it is imperative that hospitals take the necessary measures to preserve electrical power at all times. Loss of water supply or medical gasses and elevator failure do not typically occur in isolation, but are often observed in the context of power outages or natural disasters.

#### ICT failures

Hospitals have become increasingly dependent on their hospital information systems for administrative, financial, and medical operations, which directly exposes them to the possible risks associated with ICT failure. ICT failures are often associated with power failures, and can result in dysfunctional communication systems [2, 13]. A complicating factor in ICT failures is that hospitals increasingly rely on connected ICT systems, which are prone to ICT failure cascades [8]. Furthermore, cyberattacks on hospitals have been on the rise worldwide, affecting numerous hospitals in high-income countries. These include a variety of threats from brute force and denial-of-service attacks to the use of phishing and malware or social engineering methods to compromise security [6]. The health sector has now become one of the most targeted sectors globally. Disturbingly, healthcare is lagging behind other sectors in data protection [21]. The security technique emphasized most often in the literature is proper employee training, because most security breaches are caused by employees accessing malicious files. These breaches are generally not stopped by ICT security systems [22]. A devastating example is the WannaCry ransomware infection of systems in more than 80 UK hospitals in 2017, resulting in the closure of some EDs [6]. The COVID-19 pandemic caused an unprecedented strain on healthcare facilities worldwide and it accelerated the use of e-health applications. This increased dependency of telemedicine has even further increased the vulnerability of healthcare to cybersecurity issues [23].

#### Hazmat events

When not properly managed (or recognized) by on-scene first responders, the boundaries of hazardous material (hazmat) events can quickly extend to the hospital environment, causing secondary contamination. This may lead to adverse symptoms and injuries in medical personnel, or the closure or evacuation of EDs. Retrospective analyses of hazmat events in the USA from 1995-2001, 2003-2006, and 2007-2013 identified 25 incidents, including 32 injured individuals and 10 hospital evacuations. Proper decontamination procedures, proper field-to-hospital communication, and effective training can help prevent secondary contamination [10, 24, 25]. Hazmat releases and radiation accidents do not only originate from the prehospital environment, they occur within hospitals too [1]. Novel biohazards, such as hemorrhagic fevers or other emerging infectious diseases, could pose another threat to emergency care, including the risk of nosocomial outbreaks [26, 27].

#### Violence

Hospital shootings are rare compared with other forms of workplace violence, but still 154 US hospital-based shootings were identified from 2000 to 2011. The ED environment has the highest odds (29%), followed by the parking lot (23%) and patients rooms (19%). Most events involved a determined shooter with a strong motive as defined by grudge (27%) or suicide (21%). Victims were mostly perpetrators (45%) or hospital employees (20%). Cities and neighborhoods with high violence rates do not appear to be at increased risk [28].

### Natural disasters

Natural disasters are broadly classified as infectious disease outbreak, climate-related (hydrometeorologic) or geophysical, and include earthquakes, volcanic eruptions, floods, hurricanes, tornadoes, wildfires, and epidemics [1, 2, 29]. Over the last decades, the incidence of natural disasters is growing, which is mainly caused by an increase of climate-related emergencies [29, 30]. Also, the scale of disasters has expanded owing to increased rates of urbanization, environmental degradation, and intensifying climate variables [29].

#### Earthquakes

Hospitals are as vulnerable to the destructive power of earthquakes as the population they serve. Even after a moderate earthquake, hospitals are at risk for both immediate nonstructural damage that may force them to evacuate patients and the delayed discovery of structural damage resulting in permanent closure [31]. Therefore, an earthquake can simultaneously be an internal and external disaster [12, 32]. During the Northridge earthquake in 1994, 73 hospitals sustained structural damage [2]. Regulations ensure that hospitals in earthquake-prone areas are built to resist the impact of earthquakes, and thereby possibly help to prevent injuries and fatalities [32]. Evacuation and disaster recovery is often challenging due to extensive infrastructural damage.

#### Floods

Among other regions in the world, the Netherlands is susceptible to floods. It was determined that 75% of the 185 Dutch hospital locations in 2015 have a conceivable flooding risk. Remarkably, most of the vulnerable hospitals shelter the ED and emergency generators on the first floor or even in the basement [33]. Floods may arise from high tides or extreme precipitation, and have the ability to dramatically impact healthcare facilities, causing power failures, ICT disturbances, loss of water supply, destruction of essential services, and complicating hospital access [13, 16]. Likewise, tsunamis, landslides, and volcanic eruptions may cause catastrophic damage to hospitals and surrounding infrastructure.

#### Hurricanes

Hurricanes, tornadoes, and storms not only directly damage hospitals as a result of destructive winds; they tend to generate high volumes of precipitation resulting in floods. The consequences of floods are discussed above. Numerous reports exist about healthcare facilities that were incapacitated by storms [13, 16, 17]. Evacuation could be complicated because of floods as well as infrastructural damage.

#### Lightning strike

Lightning can seriously impair hospital functions, especially due to peak currents which have the ability to damage ICT networks and electronic equipment. Lightning rods can be used to divert a direct hit, but up to 50% of the current will still be spread over different conductive elements in the building. Other possibilities for protective measures are limited [34].

#### Wildfires

If wildfires occur in the vicinity of hospitals, they threaten them by fire and smoke. In 2007, a Californian hospital had to evacuate 200 patients because of wildfires. The ED remained open while the hospital was closed [35].

#### Infectious disease outbreaks

Most of the aforementioned disaster types represent acute-onset disasters with immediate consequences for acute care services. By contrast, epidemics and pandemics may have longer-lasting effects, as shown by the current COVID-19 pandemic. An infectious disease outbreak can be a global health security threat. EDs require a robust risk mitigation system and need to be in a state of constant readiness to respond to potential large-scale outbreaks [36]. Previous outbreaks and the current pandemic revealed several gaps in the pandemic preparedness of EDs, including frontline staff protection, shortages of personal protective equipment (PPE) and medical gasses, nosocomial spread, and surge capacity management [36, 37]. Actions to limit drop-out and illness among staff resulting from psychological distress are vital to secure acute care during future epidemics [37]. Large-scale outbreaks may also influence ED utilization patterns, caused by fear among the public and lockdown-mediated community effects (stay-at-home-orders, reduced traffic, school closures). Especially during the first wave of the COVID-19 pandemic, ED utilization for non-COVID emergencies was markedly reduced, which adversely impacted the economics of ED care and may also have led to negative health consequences for patients who delayed emergency care [38–40].

### Manmade disasters

Radiation accidents, chemical spills, infrastructural disasters, and acts of war or terrorism are examples of manmade disasters that may affect the ED.

#### Nuclear and radiation incidents

The consequences of major radiation accidents are invisible but may be extensive. The nuclear power plant accidents of Three Mile Island (1979), Chernobyl (1986), and Fukushima (2011) prompted the evacuation of multiple hospitals in the contaminated areas. These evacuations were characterized by fear for radiation and resulted in chaos. Following the Fukushima accident, it was reported that more than 50 patients died either during or soon after evacuation, probably due to a lack of medical support during rushed transports [15, 41]. Similarly, external fires with the release of toxic substances or chemical accidents in factories may impact nearby hospitals. Evacuation is rarely indicated, but the chemical spill may necessitate the sealing of the hospital’s ventilation ducts and/or the closure of ORs.

#### Infrastructural disasters

Infrastructural disasters are generally related to essential services. The Northeastern power outage of 2003 lasted for 38 h and affected the eastern seacoast and the northern Midwest of the USA, as well as portions of Canada. This prolonged power outage was associated with loss of communications, escalating temperatures in the hospitals, elevator failures, contaminated water supplies, and forced hospital evacuations [42]. The burst of a major water main in Amsterdam, the Netherlands, in 2015, resulted in the flood of the three lower floors of an academic hospital. The hospital’s water supply station and other vital infrastructures were severely damaged, necessitating the complete evacuation of the facility. The ED was designated as the emergency operations center where in-patients were registered, triaged, and subsequently evacuated to other hospitals [43]. The Beirut ammonium nitrate explosion in 2020 is another example of an infrastructural disaster with a major impact on healthcare. This largest non-nuclear blast in modern history resulted in three major hospitals becoming completely nonfunctional and three other hospitals sustaining partial damage [18].

#### Warfare

Although attacks on medical facilities are forbidden under international humanitarian law, these war crimes are occasionally reported. For example, during the Second Lebanon War in 2006, at least two Israeli hospitals were subjected to continuous rocket attacks. Patients and departments were rapidly relocated to previously prepared bunkers and underground shelters prior to the first rockets hit the facilities. The hospitals remained functional during the entire conflict [44, 45]. In 2015, a trauma center in Kunduz, Afghanistan, was hit by an airstrike, killing 42 people, including 14 staff members [46]. During the civil war in Syria, hospitals have been routinely targeted, and the International Committee of the Red Cross reported that more than 4200 people were victims or violence against healthcare from 2012-2014 in 11 countries [47].

#### Terrorist attacks

Hospitals could be identified as soft targets for terrorism. Between 1981 and 2013 approximately 100 terrorist attacks have been perpetrated at hospitals, in 43 countries all over the world, killing 775 people and wounding 1217 others [48]. EDs are extra vulnerable, being accessible by community members 24 h a day/7 days per week. A terrorist attack in the ED could result in staff, patients, and visitors being killed, injured, or taken hostage, and cause severe damage to the facility. Healthcare facilities should therefore also acknowledge the possibility of secondary attacks against hospitals. Secondary attacks are incidents in which an initial attack takes places elsewhere, followed by a second attack on the hospital where patients from the initial attack are being treated. Consequently, controlling ED access is a prerequisite in any hospital preparation/response plan for a terrorist attack [49]. Hojman et al. [14] described the discovery of a suspicious backpack in the ED, shortly after receiving wounded patients from the Boston Marathon bombings. The backpack was initially identified as a possible explosive device, which forced an evacuation of the ED at a time when staff was still triaging and treating the victims. Fortunately, the backpack was found not to contain any explosives. This incident yielded important lessons nonetheless: complete hospital lockdown impairs the entrance of personnel to enter the building; overflow of ED personnel exposes additional individuals to potential attacks; and ambulances were identified as a potential threat. Likewise, a study from the USA demonstrated that ambulances are susceptible to possible hijacking for terrorism purposes [50]. Access to ambulance bays should therefore be limited too [49]. During the COVID-19 pandemic, it was observed that hospitals were among the only remaining soft targets for attacks, making them increasingly vulnerable to (cyber)terrorism [23].

### Evacuation

Evacuation of critically ill patients require specialized equipment and training in order to be performed safely, especially when it involves vertical evacuation [51]. Potential relocation areas for all essential departments should be established in advance, such as within the institutions itself, to neighboring hospitals or using resources such as (military) emergency hospitals [2, 9, 52]. There are specific triage methods for evacuation (Healthcare Evacuation Reverse Triage Priorities), which may ease up the process of selection. However, there is still no common guide for evacuation, and many hospitals lack the proper preparedness [53].

## Conclusions

Hospitals and their EDs are at risk for crises and their potential escalation to hospital disasters. As hospitals evolve to better care for their citizens, the possible hazards and risk factors for IHCDs have changed over time. Emerging risks due to climate-related emergencies, infectious disease outbreaks, terrorism, and cyberattacks pose particular threats. While acute-onset disasters have immediate consequences for acute care services, epidemics and pandemics are threats that can have long-term sequelae. If hospitals are not prepared for these contingencies, patients may experience adverse outcomes as a result. Hospitals should account for regional hazards in facility construction and design. Furthermore, attention should be given to ICT resilience to prevent failures and thwart cyberattacks, and the special character of hospitals and EDs during times of crisis and disaster should be acknowledged.

## Data Availability

Not applicable.

## Abbreviations

CCTV: Closed-circuit television
ED: Emergency department
ICT: Information and communication technology
ICU: Intensive care unit
IHCDs: Internal hospital crises and disasters
